# Pterocarpanquinone LQB-118 Induces Apoptosis in *Leishmania (Viannia) braziliensis* and Controls Lesions in Infected Hamsters

**DOI:** 10.1371/journal.pone.0109672

**Published:** 2014-10-23

**Authors:** Luciana Costa, Roberta O. Pinheiro, Patrícia M. L. Dutra, Rosiane F. Santos, Edézio F. Cunha-Júnior, Eduardo C. Torres-Santos, Alcides J. M. da Silva, Paulo R. R. Costa, Silvia A. G. Da-Silva

**Affiliations:** 1 Laboratório de Imunofarmacologia Parasitária, Departamento de Microbiologia, Imunologia e Parasitologia, Universidade do Estado do Rio de Janeiro, Rio de Janeiro, Brazil; 2 Laboratório de Hanseníase, Instituto Oswaldo Cruz, FIOCRUZ, Rio de Janeiro, Brazil; 3 Laboratório de Bioquímica de Protozoários e Imunofisiologia do Exercício, Departamento de Microbiologia, Imunologia e Parasitologia, Universidade do Estado do Rio de Janeiro, Rio de Janeiro, Brazil; 4 Laboratório de Bioquímica de Tripanosomatídeos, Instituto Oswaldo Cruz, FIOCRUZ, Rio de Janeiro, Brazil; 5 Laboratório de Catálise Orgânica, Núcleo de Pesquisas de Produtos Naturais, Universidade Federal do Rio de Janeiro, Rio de Janeiro, Brazil; 6 Laboratório de Química Bioorgânica – Núcleo de Pesquisas de Produtos Naturais, Universidade Federal do Rio de Janeiro, Rio de Janeiro, Brazil; Federal Institute for Vaccines and Biomedicines, Germany

## Abstract

Previous results demonstrate that the hybrid synthetic pterocarpanquinone LQB-118 presents antileishmanial activity against *Leishmania amazonensis* in a mouse model. The aim of the present study was to use a hamster model to investigate whether LQB-118 presents antileishmanial activity against *Leishmania (Viannia) braziliensis*, which is the major *Leishmania* species related to American tegumentary leishmaniasis. The *in vitro* antileishmanial activity of LQB-118 on *L. braziliensis* was tested on the promastigote and intracellular amastigote forms. The cell death induced by LQB-118 in the *L. braziliensis* promastigotes was analyzed using an annexin V-FITC/PI kit, the oxidative stress was evaluated by 2′,7′-dichlorodihydrofluorescein diacetate (H_2_DCFDA) and the ATP content by luminescence. *In situ* labeling of DNA fragments by terminal deoxyribonucleotidyl transferase-mediated dUTP nick end labeling (TUNEL) was used to investigate apoptosis in the intracellular amastigotes. *L. braziliensis*-infected hamsters were treated from the seventh day of infection with LQB-118 administered intralesionally (26 µg/kg/day, three times a week) or orally (4,3 mg/kg/day, five times a week) for eight weeks. LQB-118 was active against the *L. braziliensis* promastigotes and intracellular amastigotes, producing IC50 (50% inhibitory concentration) values of 3,4±0,1 and 7,5±0,8 µM, respectively. LQB-118 induced promastigote phosphatidylserine externalization accompanied by increased reactive oxygen species production and ATP depletion. Intracellular amastigote DNA fragmentation was also observed, without affecting the viability of macrophages. The treatment of *L. braziliensis-*infected hamsters with LQB-118, either orally or intralesionally, was effective in the control of lesion size, parasite load and increase intradermal reaction to parasite antigen. Taken together, these results show that the antileishmanial effect of LQB-118 extends to *L. braziliensis* in the hamster model, involves the induction of parasite apoptosis and shows promising therapeutic option by oral or local routes in leishmaniasis.

## Introduction

Leishmaniases are neglected diseases that occur in 98 countries and show an annual incidence of 1 to 1.5 million people worldwide [1]–[3]. American tegumentary leishmaniasis (ATL) presents with a spectrum of clinical forms, including cutaneous, mucosal and disseminated and diffuse cutaneous leishmaniasis. *Leishmania (Viannia) braziliensis* is the important species that causes ATL and is the major agent for the mucosal forms in Brazil [4], [5]. In general, treatment failures and relapses are common for this form, leading to the mutilation or destruction of the affected nasopharyngeal area [5].

The treatment of leishmaniasis is restricted to a few extremely toxic drugs, quite costly and increasingly challenged by the development of parasite resistance to drugs [6], [7]. Moreover, the treatment is complicated by intrinsic, species-specific differences, resulting in variable drug susceptibilities in determinate geographical locations. Because of high cost or limited effectiveness, the supply of the new formulations of amphotericin B and oral miltefosine have been insufficient to meet the demand, especially in endemic regions [8]–[10]. The development of drugs that are less toxic, more effective, less costly and orally administrable is critical for the treatment of leishmaniasis in endemic countries [10].

Naphthopterocarpanquinone LQB-118 is a synthetic molecule produced through the hybridization of a naphthoquinone with a pterocarpan (isoflavonoid). Previous studies demonstrated that LQB-118 has antitumoral activity and induces apoptosis in cells derived from chronic myeloid leukemia patients [11]. Our group has recently demonstrated the antileishmanial activity of LQB-118 administered by the local or oral route in the treatment of BALB/c mice infected with *L. amazonensis*. This treatment was able to control the lesions and parasite load with the same efficacy as Glucantime, which was used as the reference drug [12].

Mice are naturally resistant to *L. braziliensis* infection and resolve the infection in approximately 5 weeks [13], [14]. The infection of golden hamsters with *L. braziliensis* results in a localized lesion and the dissemination of the parasite, which resembles the infection profile in humans [15], [16].

In the present study, we evaluated the activity of LQB-118 on *L. braziliensis* using golden hamster as the infection model. Our findings indicated that LQB-118 is therapeutically effective when administered orally or intralesionally and is active *in vitro* against *L. braziliensis*, selectively inducing DNA fragmentation in the intracellular amastigote.

## Materials and Methods

### LQB-118

LQB-118 was synthesized as previously described [17] in the Laboratory of Bioorganic Chemistry of the Federal University of Rio de Janeiro, Brazil. LQB-118 was dissolved in DMSO (dimethylsulfoxide) (Sigma Aldrich, St Louis, MO, USA).

### Parasites

*Leishmania braziliensis* (MCAN/BR/98/R619) was routinely isolated from hamster lesions and maintained as promastigotes in Schneider's insect medium (Sigma-Aldrich, St Louis, MO, USA) containing 20% heat-inactivated fetal bovine serum (Cultilab, Brazil) and 100 µg/ml gentamicin (Schering-Plough). The parasites were used after no more than five passages.

### Animals

Female golden hamsters (*Mesocricetus auratus*) six to eight weeks old were obtained from Fundação Oswaldo Cruz. This study was carried out in strict accordance with the recommendations in the Guide for the Care and Use of Laboratory Animals of the Brazilian National Council of Animal Experimentation. The protocol was approved by the Ethics Committee on Animal Use (CEUA) of the Instituto de Biologia Roberto Alcantara Gomes of the Universidade do Estado do Rio de Janeiro-UERJ, by the number protocol 044/2009.

### Antipromastigote growth

Stationary-phase, *L. braziliensis* promastigotes at 5×10^5^ cells/mL were cultured in a 24-well culture plate (1 mL/well) at 28°C in Schneider's medium plus 20% heat-inactivated fetal calf serum containing the indicated concentrations of LQB-118. The controls were treated with 0,2% DMSO, which was the highest concentration of DMSO present in the LQB-118 treatments (0–20 µM). To determine whether the antileishmanial effect of LQB-118 was reversible, the parasites were incubated with LQB-118 (0–20 µM) for 48 h at 26°C, and the number of parasites was then counted. The cells were centrifuged (1000×g for 10 min), washed twice in PBS, their number ajusted and incubated again for another 48 h at 28°C with Schneider's medium plus 20% fetal bovine serum, and then the promastigotes were counted in a Neubauer chamber.

### Antiamastigote activity

Resident macrophages were obtained from the peritoneal cells of golden hamsters after the peritoneal injection of 10 mL of DMEM. The peritoneal cells (2×10^6^/mL) were plated onto glass coverslips placed within the wells of a 24-well culture plate (0,5 mL/well) and incubated at 37°C in 5% CO_2_ for 1 h. After removing the nonadherent cells, the monolayers were infected with 5 promastigotes for each macrophage for 4 h at 37°C in 5% CO_2_. The infected macrophages were washed and incubated with several concentrations of LQB-118 for 48 h at 37°C in 5% CO_2_. The monolayers were then stained with Giemsa, and at least 100 infected macrophages per sample were counted under optical microscopy. The supernatant was collected for nitric oxide analysis. The 50% inhibitory concentration (IC50%) was determined by logarithmic regression analysis using GraphPad Prism 5.

### Determination of nitric oxide production

For the analysis of the production of nitric oxide (NO) by the infected macrophages, the culture supernatant was collected after 48 h of LQB-118 treatment, as described above. The production of NO was measured by assaying nitrite (NO_2_) using the Griess reaction, as described previously [18].

### Measurement of ROS levels in *L. braziliensis* promastigotes

Intracellular ROS levels were measured in treated and untreated cells as described previously [19]. Briefly, promastigotes of *L. braziliensis* were cultured in Schneider's insect medium supplemented with 10% HIFCS and incubated at 26°C in concentrations of LQB-118 ranging from 0 to 10.0 µM. After 48 h, the cultures were washed three times and the parasite concentration was adjusted to 1×10^7^ cells/mL. Then, 20 µM of 2′,7′-dichlorodihydrofluorescein diacetate (H_2_DCFDA) (Molecular Probes, Eugene, OR, USA) was added, and the samples were incubated for 30 min under dark conditions. The fluorescence was monitored using a Spectra Max GEMINI XPS spectrofluorometer (Molecular Devices, Silicon Valley, CA, USA) at excitation and emission wavelengths of 507 and 530 nm, respectively.

### ATP content determination

Intracellular ATP concentration was measured in treated and untreated cells using a CellTiter-Glo luminescent assay (Promega), which generates a signal proportional to the ATP amount. Briefly, promastigotes of *L. braziliensis* were cultured in Schneider's insect medium supplemented with 10% HIFCS and incubated at 26°C in the absence or presence of LQB-118 (2.5, 5, 10 and 20 µM). After 48 h, the cultures were washed three times and the parasite concentration was adjusted to 1×10^7^ cells in 200 µL of PBS. A 50 µL aliquot of each sample was transferred to a 96-well plate, mixed with the same volume of CellTiter-Glo and incubated in the dark for 10 min, and the bioluminescence was measured using a GloMax-Multi Microplate Multimode Reader (Promega). ATP concentrations were calculated from the ATP standard curve. The respiratory chain inhibitor KCN 1 mM (inhibitor of the complex IV) was used as control.

### Mitochondrial membrane potential (Ψ_m_)

To determine the effect of LQB-118 on the ΔΨ_m_ in macrophages (2×10^6^ cells/mL) were cultured in the presence of 3.125, 6.25, 12.5, 25, 50, 100 and 200 µM of LQB-118 at 37°C/5%CO_2_. After 48 h, the cells were incubated for 10 min with JC-1 (Sigma-Aldrich), washing in Hanks balanced salt solution (HBSS) and fluorescence was measured spectrofluorometrically at both 530 and 590 nm using 485 nm as the excitation wavelength. The ratio between red and green fluorescence (i.e. 590/530 nm) determined the ΔΨ_m_ [20].

### *In situ* detection of DNA fragmentation by terminal deoxynucleotidyl transferase (TdT)-mediated dUTP nick end labeling (TUNEL)

DNA fragmentation into the cell was analyzed using an *in situ* cell death detection kit (Roche) according to the manufacturer's instructions. Briefly, the hamster macrophages (4×10^5^/well) were adhered in monolayers to Lab-Tek eight-chamber slides (Nunc, Roskild, Denmark), infected (5 promastigotes per macrophage) and treated with LQB-118 for 48 h at 37°C in 5% CO_2_. The monolayers were washed twice in PBS and fixed in paraformaldehyde (4%) for 10 min. The cells were washed in PBS, incubated in a solution of 3% hydrogen peroxide and methanol for 10 min and then washed again with PBS. The monolayers were permeabilized with a solution of 0,1% Triton x-100 and 0,1% sodium citrate and then labeled with the TUNEL solution for 1 h at 37°C. Finally, the monolayers were washed with PBS and analyzed by fluorescence microscopy (Nikon Eclipse-80i).

### Flow cytometric analyses of externalized phosphatidylserine

The phosphatidylserine externalization by the parasites was assessed using an annexin V-FITC staining kit (BD Biosciences, San Diego, USA). Briefly, promastigotes (2×10^6^ cells/mL) were incubated in the absence or presence of LQB-118 (5, 10 or 20 µM) for 24 and 48 h at 28°C. The cells were washed twice and resuspended in 100 µl binding buffer (10 mM HEPES, 150 mM NaCl and 2.5 mM CaCl_2_), containing 1 µl annexin V-FITC. After 20 min, the cells were washed twice and resuspended in 300 µl of binding buffer; then, at the time of data acquisition, 1.67 µg/mL of propidium iodide was added. The data were acquired using a BD Accuri C6 flow cytometer (BD Accuri, Ann Arbor, MI, USA) and analyzed with CFlow software.

### *In vivo* activity

Golden hamsters (*Mesocricetus auratus*) were used for the infection model. The animals were held 5–6/group and infected in the hind footpad with 10^7^ stationary-phase, *L. braziliensis* promastigotes. Beginning 7 days postinfection, each group was treated for eight weeks with LQB-118 (solubilized in DMSO) administered three times a week by the intralesional route (26 µg/kg/day), LQB-118 (partially solubilized in PBS) given five times a week by the oral route (4,3 mg/kg/day) or meglumine antimoniate (Glucantime, Sanofi-Aventis) given by the intraperitoneal route (70 mg/kg/day). The groups of control animals received 10 µl DMSO (Sigma) by the intralesional route three times a week or were left untreated. The lesion sizes were measured weekly with a dial caliper (Mitutoyo, Brazil). In the eight weeak of infection, the intradermal reaction of *L. braziliensis* antigen was evaluated. The left hind footpad was injected with 20 µl of total antigen (obtained by freezing and thawing) corresponding at 2×10^6^ promastigotes. Twenty-four h later the footpad swelling was measured with dial callipper, and the intradermal reacton was expressed as the difference between thickness prior and to injection. The hamsters were euthanized at the end of the experiment using carbon dioxide chamber, and their infected paws were aseptically excised, skinned, weighted and ground in Schneider's medium (Sigma) containing 20% fetal calf serum. The cell suspension was serially diluted, and the motile parasites were evaluated using limiting dilution analysis after 7 days.

### Statistical Analysis

The statistical analyses of the *in vitro* drug effects on the promastigote and amastigote forms were performed using Student's t-test. The statistical analyses of the *in vivo* experiments were performed using analysis of variance (ANOVA) and the Tukey post hoc test.

## Results

### *In vitro* antileishmanial activity against *L. braziliensis*

**Figure 1:**
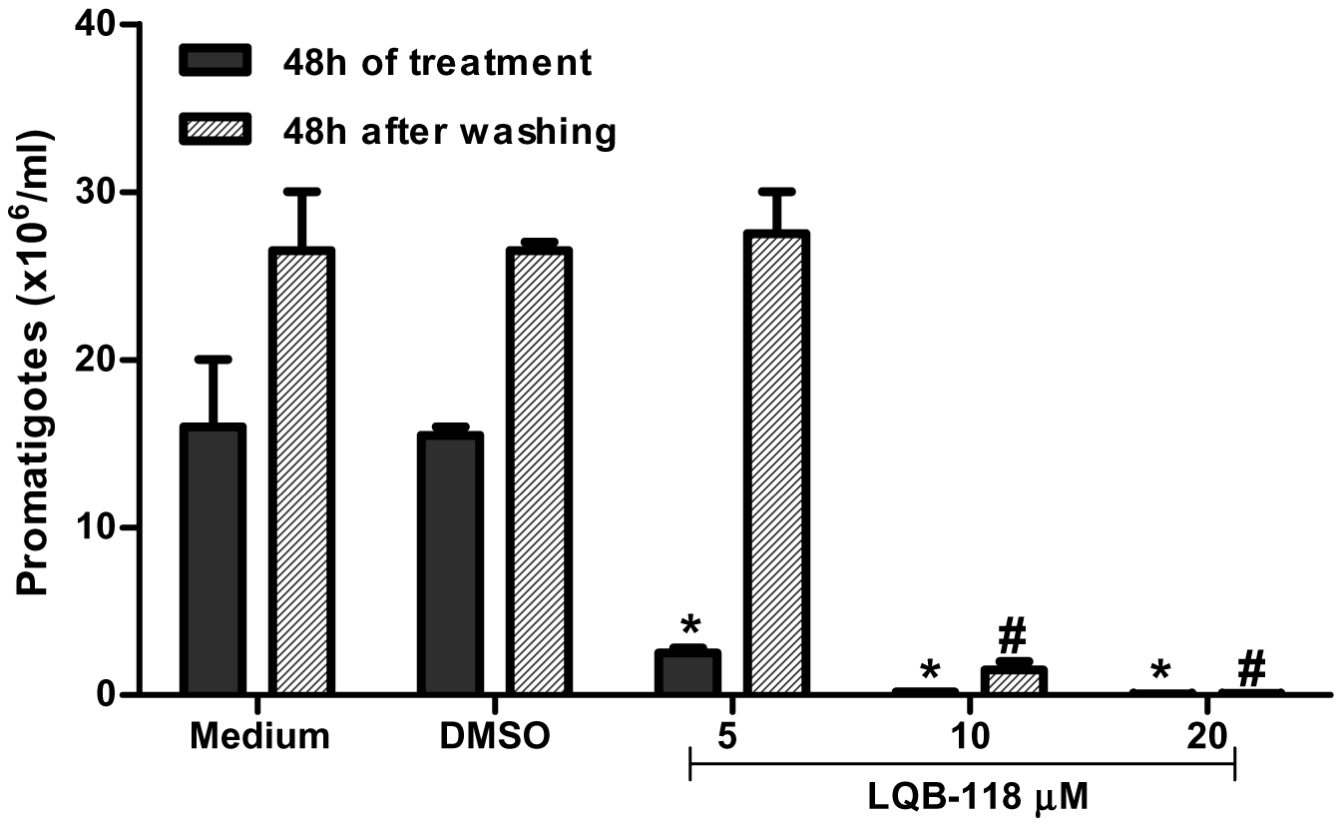
*In vitro* anti-Leishmanial effect of LQB-118 on promastigotes. Promastigote forms of *L. braziliensis* were treated with the indicated concentrations of LQB-118 for 48 h/28°C. Parasite number were counted (black bars), washed and incubated for more 48 hours at 28°C without LQB-118 (crosshatched bars). Mean ± SD, n = 3, *p<0.001 (LQB-118 treatment in relation to DMSO), # p<0.001 (LQB-118 in relation DMSO after washing and incubated).

To evaluate whether the previously demonstrated antileishmanial activity of LQB-118 on *L. amazonensis* also occurred on *L. braziliensis*, promastigotes were initially cultivated in the presence of different concentrations of LQB-118 (0–20 µM) for 3 days. LQB-118 inhibited the growth of the parasites in a dose-dependent manner and completely prevented growth at the 20 µM concentration (p<0,001) (Figure 1A, black bars). The value of the 50% inhibitory concentration (IC_50_) was estimated at 3,4±0,1 µM. To determine whether the decrease in the promastigote multiplication induced by LQB-118 was reversible, we treated the promastigotes with LQB-118 for 48 h and after this time, the cells were washed and reincubated for another 48 hours in the absence of LQB-118. The inhibitory effect on growth was not reversed when LQB-118 (at 10 or 20 µM) was removed from the culture (Figure 1, crosshatched bars).

**Figure 2:**
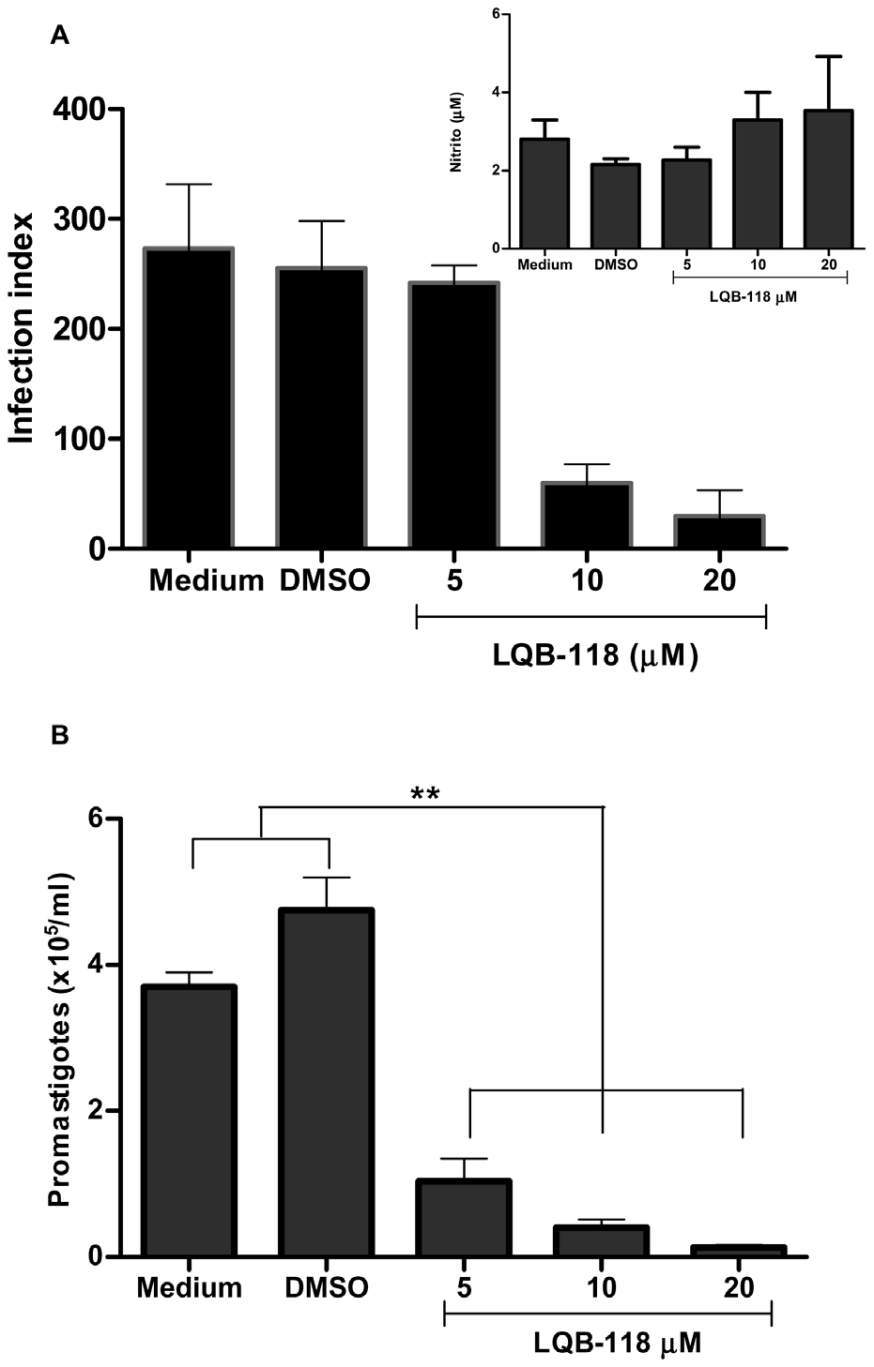
In vitro Activity of LQB-118 on *L. braziliensis*-infected macrophages. Monolayer of hamster peritoneal macrophage were infected with *L. braziliensis* (ratio of 5 parasites/macrophages) and subjected to treatment with the indicated concentrations of LQB-118 for 48 hours at 37°C/5% CO2. **A**) Macrophage monolayer was stained and the infection index was determined by counting at least 100 macrophage. Nitric oxide production in the supernatants was measured by assaying for nitrite using Griess method (inset) or **B**) After 48 h of the treatment the monolayers of infected macrophages were washed twice and incubated with Schneider's medium and 20% fetal bovine serum at 28°C for more 48 h and promastigotes were counted (mean ± SD, n = 3). * p<0, 01, ** p<0, 001(LQB-118 treatment in relation to control).

To investigate whether the antileishmanial effect of LQB-118 observed in the promastigote forms extended to the intracellular amastigote, infected hamster macrophages were treated with different LQB-118 concentrations (0–20 µM) for 48 h. Figure 2A shows a dose-dependent inhibition of the infection index (p<0.01). The IC_50_ was calculated as 7,5±0,8 µM. The nitric oxide concentration was measured in the supernatants of the cultures, and there were no changes in its production under these conditions (Figure 2A, inset). To evaluate the survival of the amastigotes remaining after LQB-118 treatment, we investigated the ability of these amastigote to differentiate into promastigotes. The monolayers of infected and treated macrophages were washed and reincubated with Schneider's medium and 20% fetal bovine serum at 28°C for another 48 hours, and the promastigotes were then counted in a Neubauer chamber (Figure 2B). The results showed that the remaining amastigotes lost the capacity to differentiate into promastigotes. Although the treatment at 5 µM did not decrease the number of intracellular amastigotes (Figure 2A), their capacity to differentiate into promastigotes was compromised (Figure 2B).

### Induction of apoptosis in *L. braziliensis* promastigotes and amastigotes by LQB-118

**Figure 3:**
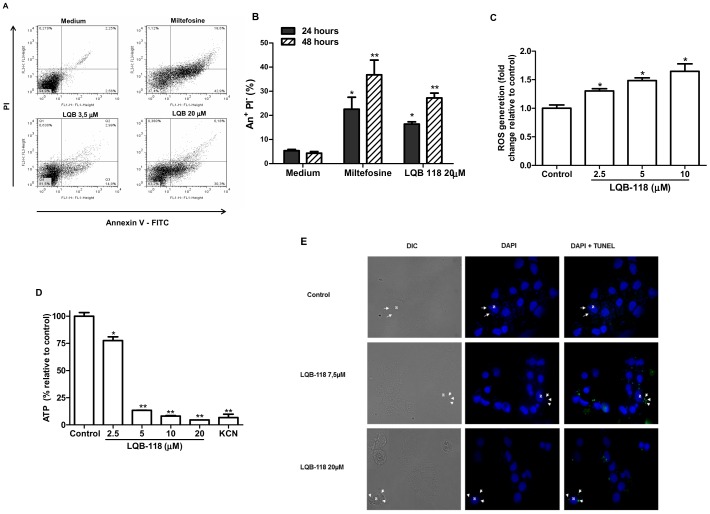
Evaluation of LQB118 inducing apoptosis on *L. braziliensis* – A) and B) Phosphatidylserine exposure on promastigotes. Promastigotes were incubated with 3,5 or 20 µM LQB118 to 24 or 48 h/28°C and then stained with Annexin V-FITC+propidium iodide and analyzed by flow cytometer. Controls were promastigotes incubated with 60 µM Miltefosine or the culture medium supplemented with 20% fetal bovine serum. In **A** only treatment at 48 h and in **B** quantitative evaluation of cells stained with annexin V-FITC at 24 and 48 h. (mean ± SD, n = 3). * P<0.05 ** P<0.01. **C) ROS generation and D) impairment of ATP production in LQB-118-treated promastigotes**. Promastigotes of *L. braziliensis* were incubated for 48 h in the presence of LQB-118 in Schneider's insect medium plus 10% HIFCS. **A**) ROS generation was quantified using H_2_DCFDA, **B**) Cellular ATP concentration was measured by bioluminescence assay. Results are presented as means ± standard error; *n* = 3. *P<0.05; **P<0.01. **E)** ***In situ*** **DNA fragmentation of intracellular amastigote**. Infected monolayers of hamsters peritoneal macrophages were treated with the indicated concentrations of LQB118 for 48 h. Monolayers were labeled using TUNEL and observated using fluorescence microscopy. Highlight the cells in 400× magnification. Legend Arrows indicate intracellular amastigotes, N – Macrophage nucleus.

To determine whether the irreversible effect of LQB-118 observed on *L. braziliensis* occurred because of the induction of apoptosis, cells treated with LQB-118 were investigated for labeling with annexin V-FITC/PI, ROS production, ATP depletion and DNA fragmentation. The promastigote forms treated with 3,5 µM (IC_50_) and 20 µM LQB-118 showed annexin labeling (AnV^+^, PI^−^) at 48 h (Figure 3A). The treatment with 20 µM LQB-118 at 24 h and 48 h promoted the labeling of 16.9% and 30.3%, respectively (Figure 3B). As oxidative stress is other hallmark of apoptosis, we also analyzed the generation of reactive oxygen species (ROS) by LQB-118-treated promastigotes. A concentration-dependent increase in ROS production was achieved in cells treated with LQB-118 (Figure 3C). Interestingly, this increase in ROS production was paralleled to a severe depletion of ATP stocks (Figure 3D). To extrapolate these findings to the clinically relevant form of the parasite, hamster peritoneal macrophages were infected with *L. braziliensis*, treated with LQB-118 and DNA fragmentation was evaluated by TUNEL assay. Fluorescence analysis revealed that 7.5 µM LQB-118 was enough to induce DNA fragmentation in intracellular amastigotes (green-labelled structures in Figure 3E) and a concentration as high as 20 µM kept the selectivity against the parasite, as no label is seen in the host cell nucleus (Figure 3E).

### Effect of LQB-118 on the mitochondrial membrane potential (ΔΨ_m_) of hamster macrophages

**Figure 4:**
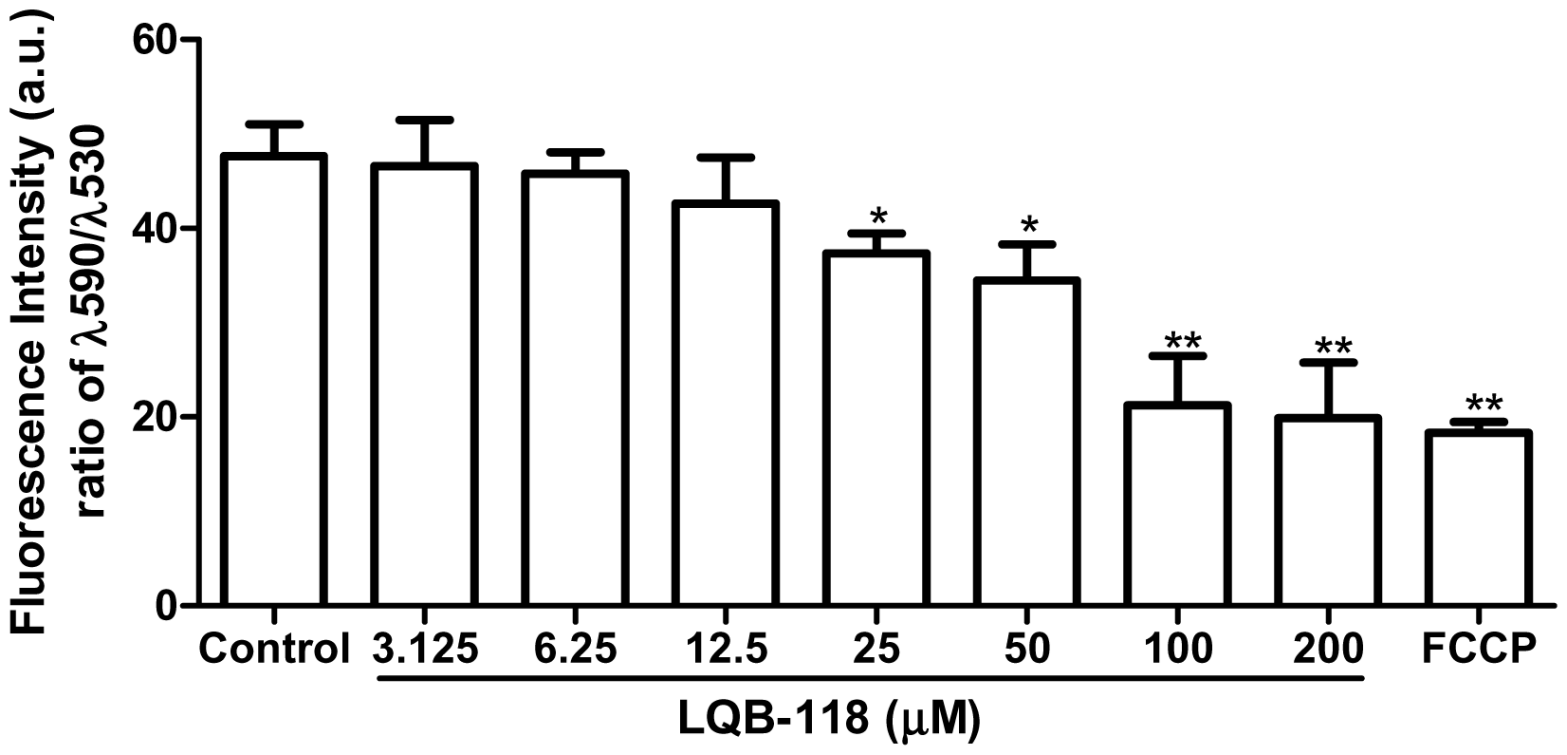
Changes in the ΔΨ_m_ of macrophage. Peritoneal hamster macrophages (2×10^6^ cells/mL) were cultured in the presence of LQB-118 for 48 h at 37°C/5%CO_2_. The cells were incubated for 10 min with JC-1 and analysed fluorometrically. Results are presented as means ± standard error; *n* = 3. *P<0.05; **P<0.01.

To evaluate the selectivity of LQB-118, we sought to investigate the mitochondrial function of LQB-118-treated hamster macrophages. In agreement with our previous results on mice macrophages (data not shown), no toxicity in hamster macrophages was observed in the IC_50_ range. Loss of ΔΨ_m_ in host cells was weakly observed just with 25 µM LQB-118. Expressive mitochondrial depolarization was obtained only in concentrations higher than 100 µM after 48 h LQB-118 treatment (Figure 4).

### Effect of LQB 118 on hamsters infected with *L. braziliensis*

**Figure 5:**
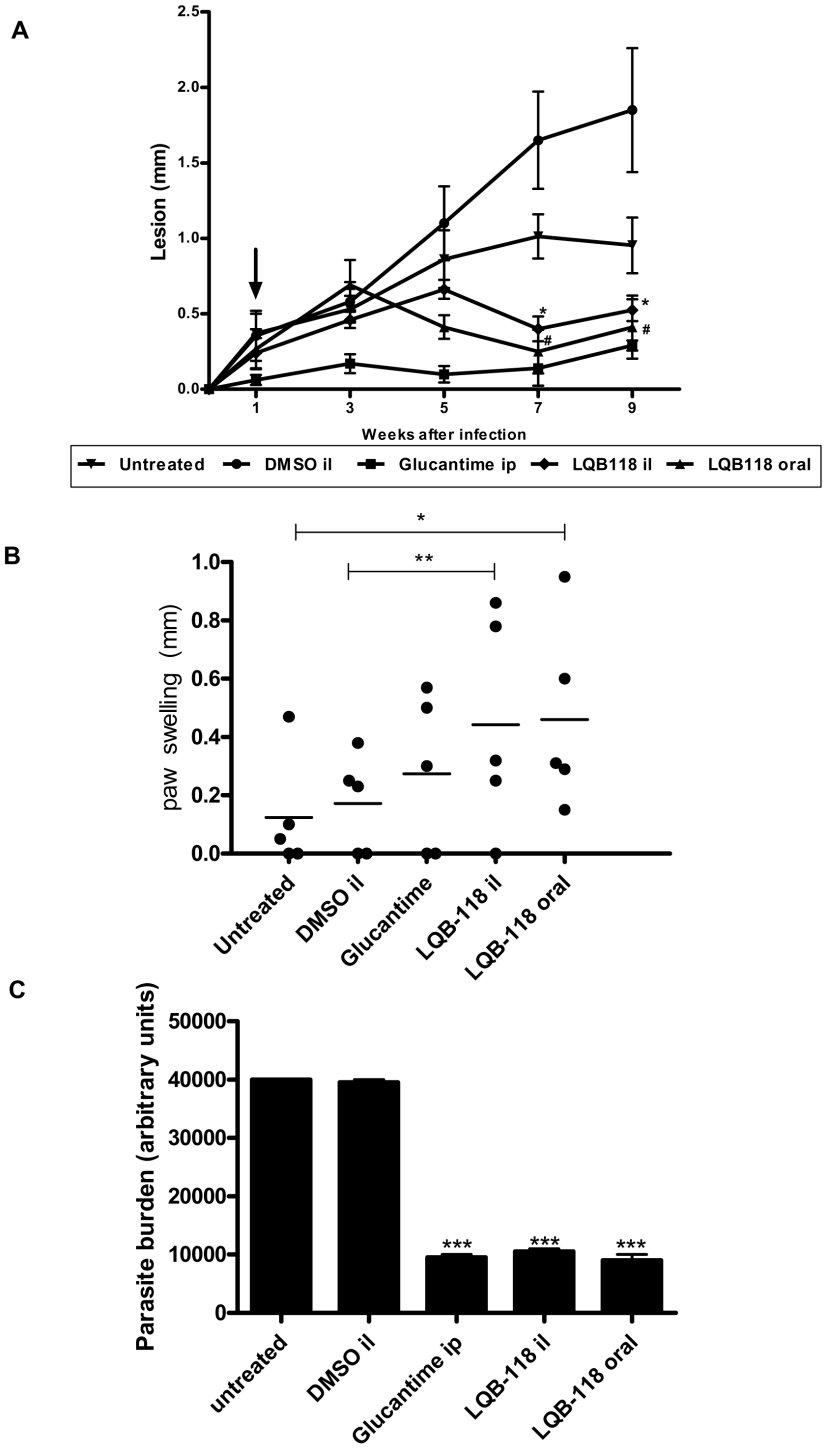
Activity of LQB-118 on golden hamsters infected with *L. braziliensis*. Golden hamsters (5/group) infected with *L. braziliensis* (10^7^) were treated on the seventh day of infection with LQB-118 intralesional (26 µg/kg/day) three times/week or orally (4,3 mg/kg/day) five times/week during eight weeks. Controls were untreated, treated with intralesional DMSO three times/week or Glucantime five times/week by intraperitoneal route. **A**) Lesion thickness was measured for nine weeks. The arrow indicates the start of treatment. Mean ± SD, # P<0.002 (in relation to untreated group). * p<0.001 (in relation to intralesional DMSO group); **B**) Intradermal reaction at *L. braziliensis* antigen was evaluated on the contralateral foot pad on eight week of infection. The swelling was measured 48 h later in the antigen-injected footpads. * p<0,04; ** p<0,01. Each point represents one animal and the horizontal bar indicates the mean. **C**) Parasite burden was assessment by limiting dilution at the end of treatment. *** p<0,001. il, intralesional/subcutaneous; ip, intraperitoneal.

The therapeutic effect of LQB-118 was evaluated in *L. braziliensis*-infected hamsters. Seven days after footpad infection, the hamsters were treated with LQB-118 by the intralesional (three times a week) or oral (five times a week) route for eight weeks. The controls were hamsters treated with intralesional DMSO (three times a week) or Glucantime (five times a week) by the intraperitoneal route or left untreated. LQB-118 decreased the lesion size from the fourth week of the treatment (fifth week of the infection) (Figure 5A). Although not statistically significant, the lesion size was decreased from the fourth week of the treatment (fifth week of the infection) in the groups treated with LQB-118 by the oral or intralesional routes when compared with the control groups (untreated or DMSO treated, respectively). In the eighth week of the LQB-118 treatments, both the oral and the intralesional routes were significantly effective in controlling the lesion size (Figure 5A) and parasite load (Figure 5C). During this period, we observed no difference between Glucantime and LQB-118 regarding either the lesion size or parasite load (Figure 5A and 5C).

To investigate whether protection promoted by LQB-118 treatment was associated with cellular immune stimulation, the antigen intradermal reaction was assessed. In the eighth week of infection (seventh week of treatment), the animals were challenged with injection of *L. braziliensis* antigen in the contralateral footpad. The intradermal reaction was measured by footpad swelling at 48 h later. As show in Figure 5B, the LQB-118 locally or orally administered was able increase intradermal response to antigen.

## Discussion

We previously demonstrated that the pterocarpanquinone LQB-118 presents antileishmanial activity against *L. (Leishmania) amazonensis in vitro* and *in vivo* using the mouse model [12]. In the present article, we used a hamster model to show that the antileishmanial effect of LQB-118 extends to another subgenus and includes *L. (Viannia) braziliensis*, the most important species causing ATL.

LQB-118 reduced the number of both the promastigote and amastigote intracellular forms of *L. braziliensis in vitro*, and this inhibitory effect on parasite growth was irreversible and occurred mainly with 10 and 20 µM LQB-118. The antiamastigote action of LQB-118 was independent of the production of nitric oxide by macrophages. These effects suggest that LQB-118 may be inducing cell death by apoptosis, as previously observed using myeloid leukemia cells, such as those from the multidrug-resistant, K562-Lucena cell line [11]. In the present work, we demonstrated that the LQB-118 treatment of the promastigotes of *L. braziliensis* induces annexin V labeling and intracellular amastigote DNA fragmentation without affecting macrophages DNA. Analysis of the mitochondrial function of LQB-118-treated macrophages showed ΔΨ_m_ reduction occurred at doses much higher than the IC_50_ for intracellular amastigotes. Although annexin labeling is not exclusive to the externalized phosphatidylserine (PS) membrane phospholipids of *Leishmania* [21], we additionally observed promastigote DNA fragmentation (data not shown), ROS generation and ATP production impairment in promastigotes. The increase in ROS production and ATP stocks depletion suggest mitochondrion dysfunction, which could culminate with cell death by apoptosis, as demonstrated by PS exposure and DNA fragmentation. These effects of LQB-118 on *L. braziliensis* are consistent with our recent finding concerning the cell death mechanism of LQB-118 in *L. amazonensis*. LQB-118 kills the promastigote of *L. amazonensis* by apoptosis, which induces ROS production, oxidative stress, depolarization of mitochondrial membrane potential and reduction of ATP [22]. These results suggest LQB-118 induces mitochondrion-dependent apoptosis in *Leishmania* spp. On the other hand, LQB-118-induced apoptosis in tumor cells is believed to involve different routes, depending on the cell type: increasing ROS, intracellular calcium levels and the activity of caspase 9 or 12 [23].

The golden hamster is susceptible to *L. braziliensis* and reproduces the human infection, with progressive swelling and ulceration at the inoculation site that is followed by dissemination of the parasites [15], [16]. The treatment of infected golden hamsters with LQB-118 was administered intralesionally and orally for eight weeks and significantly diminished the lesion size and parasite burden when administered by either means. The LQB-118 treatment was able promote increased of intradermal reaction to *L. braziliensis* antigen suggesting protective immune response. Intradermal reaction skin test (delayed type hypersensitivity) with *Leishmania* antigen is a good marker of cellular immune response in leishmaniais e predictive of protection [24], [25].

The lesion sizes were highly significantly (p<0,001) reduced by the intralesional treatment with LQB-118 in relation to the DMSO control group. DMSO was used to solubilize the LQB-118 doses administered by the intralesional route, but DMSO alone resulted in an increased paw size in relation to that of the untreated control group. Certain pharmacological activities are attributed to DMSO, and because of its anti-inflammatory activity, the use of DMSO has been suggested in several human diseases, including gastrointestinal and dermatologic disorders and brain edema [26]. In mice, intralesional DMSO treatment increased the paw edema induced by zimozan, whereas oral treatment had the opposite effect [27]. Because there was no significant parasitic load difference between the DMSO and untreated control groups, the DMSO most likely only induced paw swelling. Despite the increased paw swelling with the intralesional injection of DMSO, LQB-118 demonstrated a potent therapeutic action even when solubilized in this vehicle. Whether LQB-118 provides any additional anti-inflammatory effect is a question that requires further investigation. We observed no significant difference in effectiveness between the oral and intralesional routes of administration. Although the orally administered LQB-118 was partially solubilized in PBS, it was effective in the control of lesion size and parasite burden in relation to the untreated group (p<0,002 in the seventh week postinfection), showing that pterocarpanquinone is also active by this important route. Likewise, LQB-118 was orally active in controlling lesions in *L. amazonensis*-infected BALB/c mice [12]. Although oral miltefosine has been a breakthrough for leishmaniasis therapy, the drug resistance and reduced sensitivity of the parasite observed in New World species are factors limiting its use against ATL [28]. The development of new antileishmanial molecules that are orally bioavailable is an important strategy in leishmaniasis control [10]. Together, the results of our study showed that the antileishmanial effect of LQB-118 extends to *L. braziliensis* in the hamster model and is related to the induction of apoptotic cell death in the parasites. These data are an important contribution to the preclinical studies of LQB-118, demonstrating the promising therapeutic action of this new molecule on leishmaniasis.
